# Supplementary material for: High-Throughput Analysis of Neutrophil Extracellular Trap Levels in Subtypes of People with Type 1 Diabetes
Source: Biology (Basel). 2023 Jun 19;12(6):882. doi: 10.3390/biology12060882 (PMC10294788; doi:10.3390/biology12060882)
Supplement: Supplementary file 1 [file biology-12-00882-s001.zip › PROOFREAD - Supplementary figures - Biology - NETs and type 1 diabetes.pdf]

## Supplementary Figures

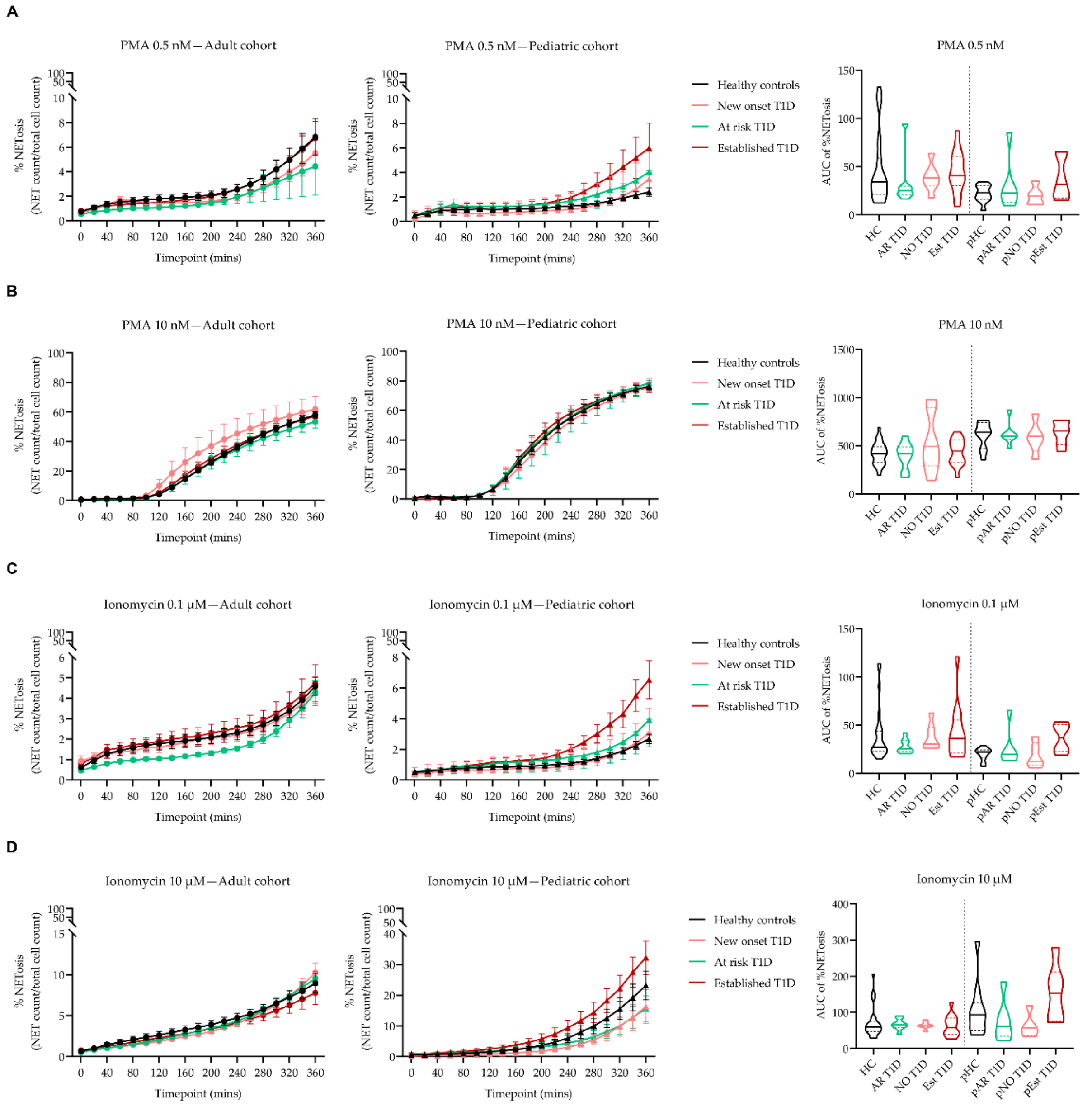


Supplementary Figure S1: Using Incucyte® ZOOM to determine NET levels in adult and pediatric T1D and HC donors. Graphs show levels phorbol-myristate acetate - (PMA, A, B) and ionomycin- (C, D) stimulated NETosis in adult (left panel) and pediatric (middle panel) T1D and HC donors across multiple timepoints (20-minute intervals). (A, B, C, D) Area under the curve (AUC) values of the graphs are represented (right panel). T1D donors are separated by subtype (new onset, at risk and established) according to the stage of disease development. Unpaired Kruskal-Wallis test with Bonferroni correction. Abbreviations: HC: healthy control; AR: at risk; NO: new onset; Est: established; pHC: pediatric healthy control; pAR: pediatric at risk; pNO: pediatric new onset; pEst: pediatric established.

Supplementary Video S1: NETosis induced by PMA- stimulation. Healthy control neutrophils were stimulated with 1 nM of phorbol-myristate acetate (PMA), stained with live (red, NUCLEAR-ID® red DNA stain) and dead (green, SYTOX® green) cell dyes and imaged at 20-minute intervals for 6 hours. Images feature the phase, red (300ms-exposure) and green (800-ms exposure) channels. Scale bar = 200 μm.

Supplementary Video S2: NETosis induced by ionomycin- stimulation. Healthy control neutrophils were stimulated with 1 μM of ionomycin, stained with live (red, NUCLEAR-ID® red DNA stain) and dead (green,

SYTOX® green) cell dyes and imaged at 20-minute intervals for 6 hours. Images feature the phase, red (300ms-exposure) and green (800-ms exposure) channels. Scale bar = 200 µm.
